# Digital PCR Modeling for Maximal Sensitivity, Dynamic Range and Measurement Precision

**DOI:** 10.1371/journal.pone.0118833

**Published:** 2015-03-25

**Authors:** Nivedita Majumdar, Thomas Wessel, Jeffrey Marks

**Affiliations:** Thermo Fisher Scientific, South San Francisco, CA, 94080, United States of America; Universidade Federal do Rio Grande do Sul, BRAZIL

## Abstract

The great promise of digital PCR is the potential for unparalleled precision enabling accurate measurements for genetic quantification. A challenge associated with digital PCR experiments, when testing unknown samples, is to perform experiments at dilutions allowing the detection of one or more targets of interest at a desired level of precision. While theory states that optimal precision (P_o_) is achieved by targeting ~1.59 mean copies per partition (λ), and that dynamic range (R) includes the space spanning one positive (λ_L_) to one negative (λ_U_) result from the total number of partitions (n), these results are tempered for the practitioner seeking to construct digital PCR experiments in the laboratory. A mathematical framework is presented elucidating the relationships between precision, dynamic range, number of partitions, interrogated volume, and sensitivity in digital PCR. The impact that false reaction calls and volumetric variation have on sensitivity and precision is next considered. The resultant effects on sensitivity and precision are established via Monte Carlo simulations reflecting the real-world likelihood of encountering such scenarios in the laboratory. The simulations provide insight to the practitioner on how to adapt experimental loading concentrations to counteract any one of these conditions. The framework is augmented with a method of extending the dynamic range of digital PCR, with and without increasing n, via the use of dilutions. An example experiment demonstrating the capabilities of the framework is presented enabling detection across 3.33 logs of starting copy concentration.

## Introduction

The digital method distributes target molecules into a large number of partitions such that each partition gets a number of molecules (0, 1, 2, etc.) theoretically following a Poisson distribution. Performing PCR on these partitions results in amplification being detected (positives) in those partitions containing one or more target molecules and no amplification being detected (negatives) in those partitions containing zero molecules.

Of interest to the investigator utilizing digital PCR techniques [1] are precision, dynamic range, and sensitivity of their digital PCR system. Digital PCR experiments resulting in the production of all positive or all negative results do not typically provide sufficient information regarding the investigated samples in question (i.e., the results are outside of the digital PCR’s effective dynamic range). Alternatively, while it is well known [2] and [3] that maximal precision is achieved at ~1.59 copies/partitions, the practical use of this knowledge is of little value to the investigator seeking to determine concentration of an unknown sample.

The relationships between digital PCR capabilities, along with the interaction of system related characteristics (available partitions and interrogated volumes) are discussed in the following section on Poisson modeling of digital PCR systems. Augmenting the theoretical aspects is a section on the practical effects on digital PCR capabilities due to conditions (false reaction calls, volumetric variability) likely to be encountered by the investigator. A method for efficiently optimizing specific digital PCR capabilities is then presented within the context of an example experiment.

## Materials and Methods

### A Poisson Model for Digital PCR Systems

As positives may contain more than one copy of the target molecule, a simple summing of the number of positives will not yield the correct number of target molecules present across the partitions. Therefore, Poisson statistics are employed to correctly estimate the total number of target molecules within the interrogated sample. This strategy presumes that every molecule has an equal chance of landing in any one of the available partitions.

The parameter λ (drawn from Poisson distribution terminology) is defined as the mean copies per partition. Equation 1 gives the maximum likelihood estimator for λ in terms of the number of negative partitions (z) and the number of partitions (n). An alternate expression is included utilizing the fraction of negative partitions (q). The MIQE guidelines for digital PCR derive these equations using the number of positive partitions [4]. If it is more convenient to count the number of positive partitions for a system, the number of negative partitions can be computed by subtracting the number of positive partitions from the total number of partitions.

$$\lambda =-\text{ln}\left(\frac{z}{n}\right)=-\text{ln}q$$ 1

Using λ from equation 1, the target concentration C is estimated by equation 2, where V is the volume trapped in each partition.

$$C=\frac{\lambda }{V}$$ 2

Within this context, the dynamic range (R) defines the span of detectable concentrations in log_10_ units and is given as

$$R=\log_{10}\frac{\lambda_{u}}{\lambda_{l}}$$ 3

The upper and lower sensitivity limits (λ_u_ and λ_l_) can be either arbitrarily determined based upon characteristic of the digital system or defined per the detection precision requirement.

Precision here is defined as the spread of the confidence interval around λ compared to the true value of λ

$$P=\frac{\max\left(\left|\lambda -\lambda_{CI_{U}}\right|,\left|\lambda -\lambda_{CI_{L}}\right|\right)}{\lambda }$$ 4

The confidence interval around the Poisson parameter λ is determined using a formalism adapted from Mather [2] on quantifying targets using dilution assays. Mather uses the fact that the variance of the maximum likelihood estimator is the inverse of the Fisher information metric in finding an expression for the standard deviation for the transformed variable ln(λ). This standard deviation (σ), given as

$$\sigma =\frac{\sqrt{1-q}}{\text{ln}q\sqrt{nq}}$$ 5

is used to estimate the confidence interval in log normal space as $CI=\bar{x}+Z\sigma$.

Exponentiating the expression for confidence intervals for the copies per reaction produces equations 6 and 7. For a 95% confidence, use Z = 1.96.

$$\lambda_{CI_{L}}=e^{\text{ln}\left(\lambda \right)+1.96\frac{\sqrt{1-q}}{\text{ln}q\sqrt{nq}}}=e^{\text{ln}\left(\lambda \right)-1.96\frac{\sqrt{e^{\lambda }-1}}{\lambda \sqrt{n}}}=\lambda *e^{-1.96\frac{\sqrt{e^{\lambda }-1}}{\lambda \sqrt{n}}}$$ 6

$$\lambda_{CI_{U}}=e^{\text{ln}\left(\lambda \right)-1.96\frac{\sqrt{1-q}}{\text{ln}q\sqrt{nq}}}=e^{\text{ln}\left(\lambda \right)+1.96\frac{\sqrt{e^{\lambda }-1}}{\lambda \sqrt{n}}}=\lambda *e^{1.96\frac{\sqrt{e^{\lambda }-1}}{\lambda \sqrt{n}}}$$ 7

Note that this estimate for the confidence interval is asymmetric. For estimating precision, we conservatively use the larger deviation from λ. Substituting equations 6 and 7 into equation 4 produces a new equation for precision expressed as follows:

$$P=\frac{\max\left(\lambda -\lambda *e^{-1.96\frac{\sqrt{e^{\lambda }-1}}{\lambda \sqrt{n}}},\lambda *e^{1.96\frac{\sqrt{e^{\lambda }-1}}{\lambda \sqrt{n}}}-\lambda \right)}{\lambda }=\max\left(\left|1-e^{\pm 1.96\frac{\sqrt{e^{\lambda }-1}}{\lambda \sqrt{n}}}\right|\right)$$ 8

As precision has the square root of n in the denominator, precision is influenced by the number of available partitions. Higher n produces smaller (better) numerical values for precision.

**Fig 1:**
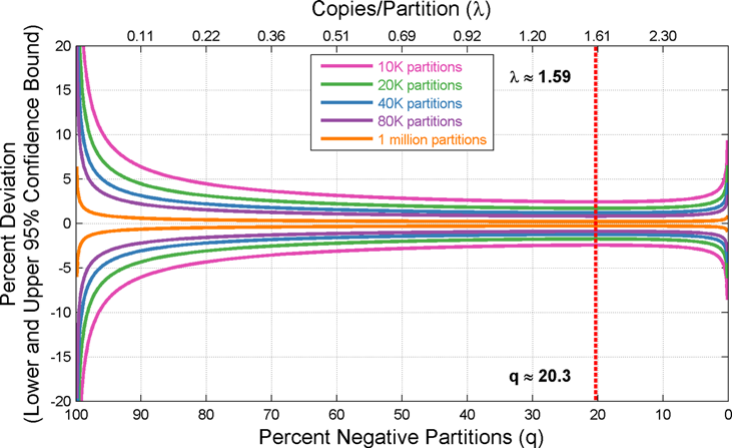
Optimal Concentration Range for digital PCR Experiments. Theoretical confidence intervals around λ across a variety of increasing available partitions. The dotted red line indicates the point of greatest achievable precision (independent of the number of available partitions) where approximately 20.3% of available partitions are negative.

Measurement precision is not uniform across concentrations. Fig. 1 shows the theoretical confidence interval around the measured value for a range of concentrations across an increasing number of available partitions. The dotted red line indicates the point of greatest achievable precision where ~20.3% of the available partitions are found to be negative. Note that the location of greatest precision is independent of the number of available partitions. Fig. 1 also shows that precision deteriorates more rapidly towards higher concentrations. From this perspective, it is preferable to error on the side of using more dilute samples when running dPCR experiments.

### Dynamic Range Dependence on Precision

Theoretical dynamic range ($\hat{R}$) is obtained between concentrations producing at least one positive to at least one negative result. With this definition, sensitivity limits (expressed as λ_î_ and λ_û_) are

λl^=−ln(1−α)1n 9

λu^=−ln(1−(1−α)1n) 10

where 1−*α* is the desired confidence level of detection. (See S1 Appendix for a derivation of these equations treating dPCR experiments as a binomially distributed random variable.)

While using these sensitivity limits produces a maximal theoretical dynamic range, the measurement variability at both extremes of concentration (nearly all negative or nearly all positive) is very high. This variability translates into a significantly reduced level of precision at these concentrations.

**Fig 2:**
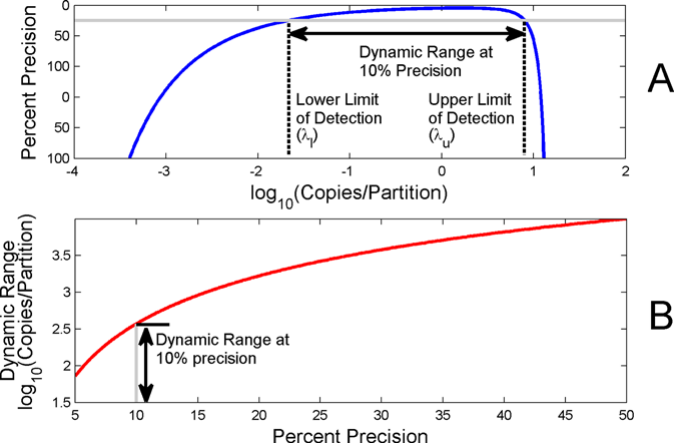
Dynamic Range dependence on Precision. Fig. 2A shows the dynamic range associated with a level of precision of at least 10% for 20K partitions. Fig. 2B shows how the dynamic range varies as the required level of precision is relaxed (moved right along x-axis) or made more stringent (moved left along x-axis).

Setting a criterion for a required level of precision (i.e., employing λ_l_ and λ_u_ from equations 6 and 7) effectively acts as a constraint on the dynamic range (*R*) but ensures a minimum level of precision across that range. Fig. 2A shows dynamic range associated with a level of precision of at least 10% (20K partitions). Fig. 2B shows how dynamic range increases or decreases (y axis) as the required level of precision changes (x axis).

### Measurement Precision Dependence on Available Partitions

**Fig 3:**
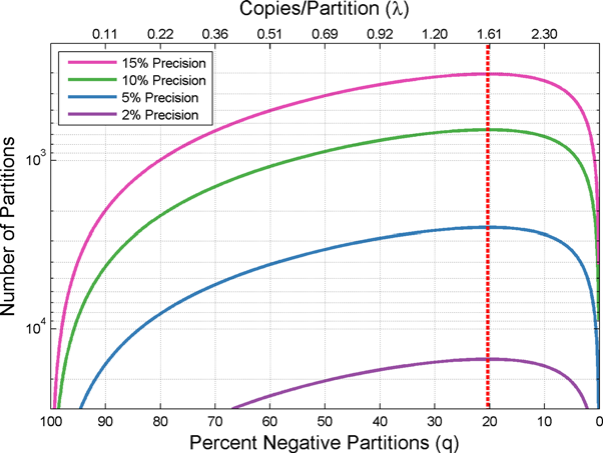
Measurement Precision dependence on Available Partitions. Measurement precision depends on the number of available partitions. Contour lines represent a specific level of precision and relate the number of partitions required to achieve the specified precision at various concentrations. The dotted red line (at approximately 20.3% negative partitions) highlights the concentration at which a minimal number of partitions are needed to achieve a particular level of precision.

Fig. 3 shows how the number of available partitions impacts measurement precision. Contour lines represent constant levels of precision when relating the number of available partitions to sample concentration. For a given concentration (selected x-axis value), changing the number of available partitions (movement along y-axis) changes the measurement precision achievable. This notion is further reinforced by noting that the minimum number of partitions needed to achieve any particular level of precision is again found to be where approximately 20.3% of the available partitions are negative (dotted red line).

### Sensitivity Dependence on Interrogated Volume

**Fig 4:**
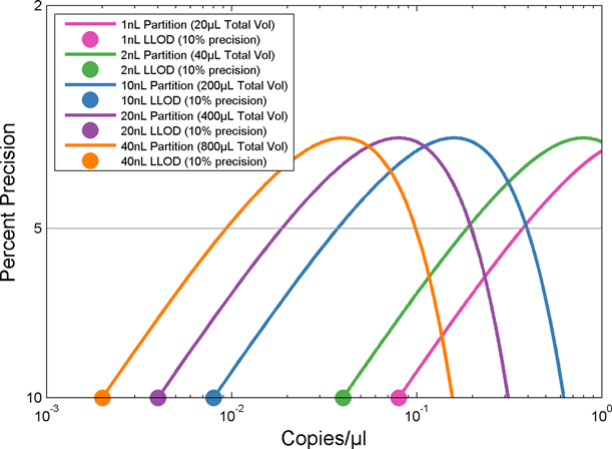
Lower Limit of Detection Dependence on Total Interrogated Volume. Total interrogated volume influences the lower limit of detection for a given number of partitions. In the plot, precision curves for a given number of partitions (20,000) are shown where the difference is in the size of the partitions (1, 2, 10, 20, and 40 nanoliters). The larger the total interrogated volume increases (due to increase in partition size), the smaller the lower limit of detection. The colored dots represent the lower limit of detection for the various partition sizes at 10% precision.

The lowest detectable concentration is almost exclusively dependent upon the total sample volume interrogated by the digital PCR system. Fig. 4 shows how the lowest detectable concentration (leftmost point on precision curves) varies as the total volume interrogated changes (assuming a fixed number of partitions). The principle contributing factor toward improved lower limit of detection is the total sample volume interrogated. For detecting rare events, the focus should thus be towards higher total sample volume than the number of partitions.

### Practical Effects on Digital PCR Capabilities

#### Impact of False Reaction Calls

A partition without a target molecule which is detected (i.e., called positive) produces a Type I error or False Positive. A partition with a target molecule which is undetected (i.e., called negative) produces a Type II error or False Negative. Possible causes for False Positives include contamination of a partition. Possible causes for False Negatives include amplification failure. Other sources of noise contributing factors include (but are not limited to) pipette errors, chemistry interaction, optical or system noise, and source sample related effects.

The following enhanced version of the digital PCR model considers probability of false reaction calls and adds an additional random noise budget to more accurately model the system.

Let the variations represented by σ correspond to the Poisson or sampling related component as shown in equation 5. Additional variation is estimated as follows: Let λ_𝜖_ denote the λ observed due to false reaction calls. This λ_𝜖_ is related to the true λ as

$$\lambda_{𝜖}=-\text{ln}\left(e^{\lambda }-{𝜖}_{+}+{𝜖}_{-}\right)$$ 11

where 𝜖_+_ and 𝜖_-_ are the false positive and false negative error rates. The fraction of negatives is thus given by

$$q_{𝜖}=e^{-\lambda_{𝜖}}$$ 12

Using the fraction of negatives given in equation 12 in equation 5, confidence bounds are given by

$$\lambda_{l_{𝜖}}=e^{\text{ln}\left(-\text{ln}q_{𝜖}\right)+1.96\frac{\sqrt{1-q_{𝜖}}}{\text{ln}q_{𝜖}\sqrt{nq_{𝜖}}}}=e^{\text{ln}\left(\lambda_{𝜖}\right)-1.96\frac{\sqrt{e^{\lambda_{𝜖}}-1}}{\lambda_{𝜖}\sqrt{n}}}=\lambda_{𝜖}*e^{-1.96\frac{\sqrt{e^{\lambda_{𝜖}}-1}}{\lambda_{𝜖}\sqrt{n}}}$$ 13

$$\lambda_{u_{𝜖}}=e^{\text{ln}\left(-\text{ln}q_{𝜖}\right)-1.96\frac{\sqrt{1-q_{𝜖}}}{\text{ln}q_{𝜖}\sqrt{nq_{𝜖}}}}=e^{\text{ln}\left(\lambda_{𝜖}\right)+1.96\frac{\sqrt{e^{\lambda_{𝜖}}-1}}{\lambda_{𝜖}\sqrt{n}}}=\lambda_{𝜖}*e^{1.96\frac{\sqrt{e^{\lambda_{𝜖}}-1}}{\lambda_{𝜖}\sqrt{n}}}$$ 14

The variation from sampling and non-zero false reaction call error rates is

$$\sigma_{𝜖}=\frac{\sqrt{e^{\lambda_{𝜖}}-1}}{\lambda_{𝜖}\sqrt{n}}$$ 15

An arbitrary source of variation related to system noise, σ_s_, is pooled along with the variation attributable to sampling and non-zero error rates, giving the total variation as follows:

$$\sigma_{t}=\sqrt{\sigma_{𝜖}^{2}+\sigma_{s}^{2}}$$ 16

This leads to an expanded confidence bound given as follows:

$$\lambda_{l_{t}}=\lambda_{𝜖}*e^{-1.96\sigma_{t}}$$ 17

$$\lambda_{u_{t}}=\lambda_{𝜖}*e^{1.96\sigma_{t}}$$ 18

Expressions 17 and 18 are substituted into equation 8 producing a more accurate estimation of precision

$$P=\max\left(\left|1-\frac{\lambda_{𝜖}}{\lambda }e^{\pm 1.96\sigma_{t}}\right|\right)$$ 19

**Fig 5:**
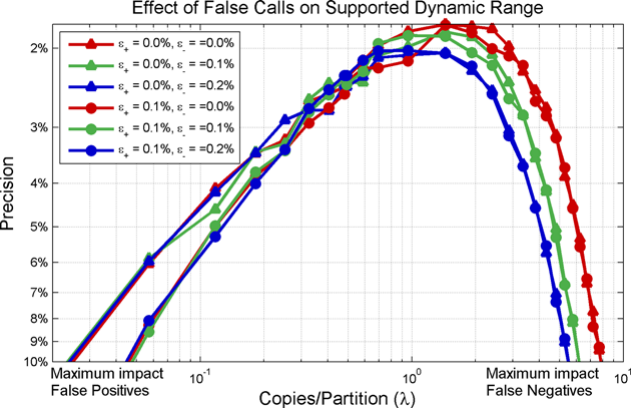
Effect of False Reaction Calls on Precision and Dynamic Range. False positives effect precision curves differently from false negatives. False positive rates are represented in the plot via shapes (triangles = 0.0%, circles = 0.1%). False negative rates are represented by colors (red = 0.0%, green = 0.1%, blue = 0.2%). Note that false positives strongly impact precision at lower concentrations (where there are very few positives) and false negatives strongly impact precision at higher concentrations (where there are very few negatives)

The validity of equation 19 using the pooled estimate for standard deviation combining the effects of sampling error, error rates and system noise is established using Monte Carlo simulations. Fig. 5 shows the effect of different false reaction call error rates on measurement precision with 𝜖_+_ indicating false positive error rate (where a percentage of simulated negative partitions are flipped to positive) and 𝜖_-_ indicating false negative error rate (where a percentage of simulated positive partitions are flipped to negative).

**Fig 6:**
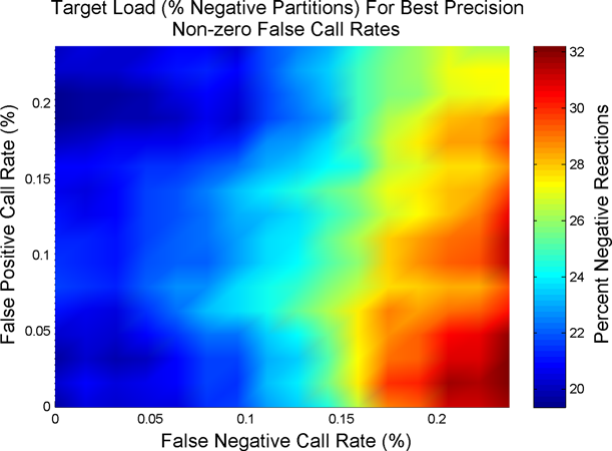
Target Load in Percent Negatives for Optimal Precision Under Non-zero False Call Rates. Optimal load concentrations under the likely scenario of non-zero false reaction call rates will no longer target 20.3% negative partitions. For example, as the false negative rate increases (with a constant false positive rate), one should target a percentage of negatives > 20.3% in order to approach optimal precision.

Fig. 6 provides a notion on how the optimal targeted copies per partition changes as the systems is influenced by non-zero false reaction call rates. Under the influence of false calling rates, the original 20.3% negative partition rate may no longer produces optimal precision. As the false negative rate increases, it is actually desirable to target a percentage of negative partitions > 20.3% for optimal measurement precision.

**Fig 7:**
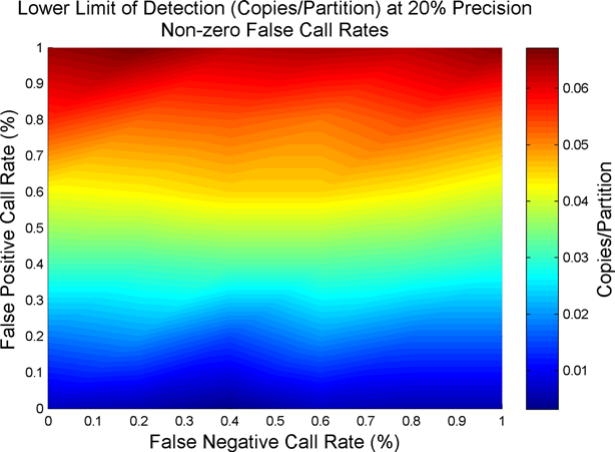
Lower Limit of Detection dependence on False Positive Rate. Lower limit of detection is influenced most heavily by false positives. This dependence is due to limited effect false negatives have in counter balancing false positives due to the limited number of positives at lower concentrations. The plot demonstrates the effects of false reaction call rates on the lower limit of detection for 20,000 partitions at 20% precision. Using a baseline of 0% false positives and 0% false negatives, the lower limit of detection is measured at 0.006 copies/partition. Raising the false positive rate to 1% results in the raising of the lower limit of detection to 0.065 copies/partition, over an order of magnitude elevation.

Fig. 7 demonstrates that as the false positive rate increases, the sensitivity at the lowest limit of detection also increases. For tracking the lowest limit of detection, the concentration of interest is where most wells are negative. On average for 20K wells, X% false positives would mean X*20,000/100 positives. A 1% false positive rate results in about 200 false positives. False negatives could be expected to counterbalance the effect of the false positives. However, these false negatives rates are not high enough to really impact the few positives that go toward determining the lowest limit of detection.

### Impact of Volume Variability

Poisson statistics are founded on the notion that the probability of any event occurring within a bounded area depends only upon the size of the area itself. Digital PCR systems, by their very nature, divide interrogated samples into a set of smaller partitions. It is common practice to make the assumption of monodisperse partitioning [5], [6], [7]. This assumption allows for the simplification of assigning a common probability of acquiring any given target molecule to each of the partitions. The impacts of volume variability in the partitioning (i.e., the effects of not making the previous simplification) are now considered.

**Fig 8:**
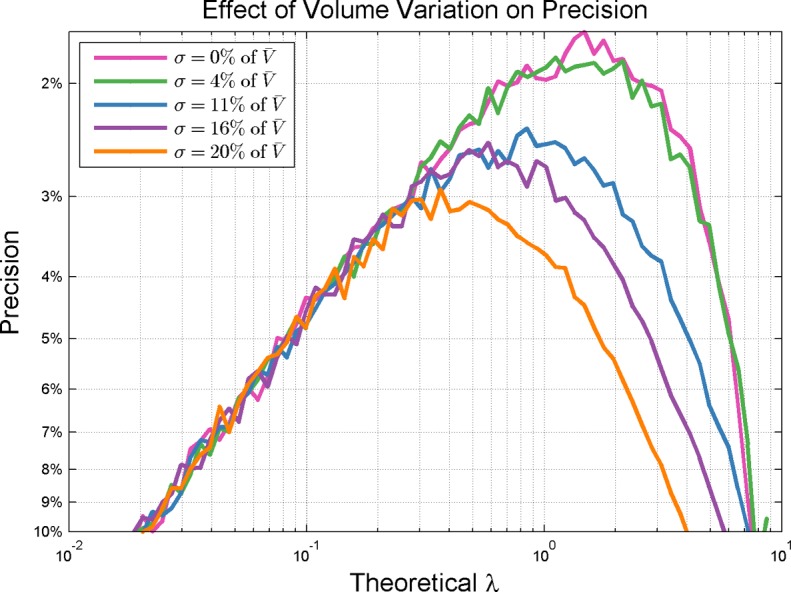
Effect of Volume Variation on Precision. Volume variation affects precision significantly at higher concentrations. Volume variability is simulated by assuming a normal distribution of well volumes with the standard deviation taken as a percentage of the mean well volume. Volumes of 865pl for 10,000 partitions were used in the simulation. Note that concentration at peak precision moves toward lower concentrations (increasing negative percentage) as volume variability increases.

The effect of volume variation among reaction chambers on estimating concentration was investigated with Monte Carlo simulations. In this simulation, the probability of a partition containing a particular molecule is proportional to the volume of the partition. A normal distribution of volume variation is assumed with the standard deviation taken as a percentage of the mean volume. Fig. 8 show that volume variation impacts higher concentrations more significantly than lower concentrations. Fig. 8 shows the percent negatives with best measurement precision under assumption of varying degrees of volume variation. It is seen that peak precision moves towards lower concentrations (i.e., increasing negative percentage) as the amount of volume variability increases.

### Efficiently Optimizing Specific Digital PCR Capabilities via Dilutions

**Fig 9:**
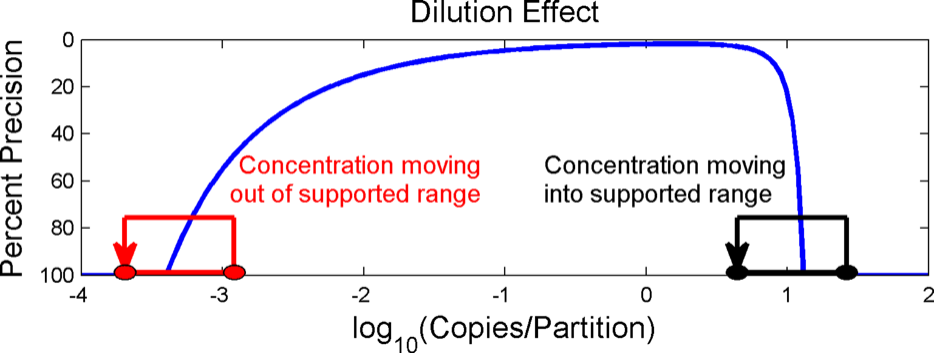
Effect of Dilutions on the Ability to Detect Concentrations Near the Limit of Detection. Diluting samples can bring samples with concentrations above the upper limit of detection into the support dynamic range of the digital PCR system. However, such dilutions can also move samples with concentrations close to the lower limit of detection outside of the supported dynamic range.

Running more than one sample concentration can extend the supported dynamic range (at a specified level of precision) for a digital PCR detection system. For sample concentrations that are higher than the supported dynamic range, dilutions can bring the concentration of the sample within the detection region. However, such dilutions might also move rare target samples below the lower limit of detection and outside the supported dynamic range (Fig. 9). To ensure the digital PCR system’s ability to accurately detect both levels of expression, it is advised to utilize both the original and diluted samples wherein the diluted sample extends the supported dynamic range while the original sample preserves the ability to detect rare targets.

**Fig 10:**
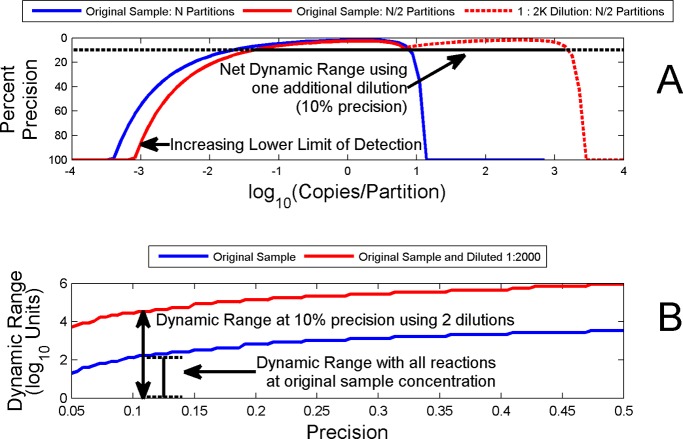
Dilution Effects on Dynamic Range and Limits of Detection—Constant Available Partitioning. Fig. shows the benefits and disadvantages of devoting a portion of available partitions to optimized dilution steps. Optimizing the dilution step is necessary to achieve a maximal increase in dynamic range while maintaining a continuous ability to detect concentrations throughout the dynamic range (i.e., without creating detection “gaps” between the steps). Fig. A demonstrates how a single dilution (using half of the available partitions) both raises the lower limit of detection on the undiluted sample and reduces the dynamic range of each individual step while increasing the overall dynamic range. Fig. B demonstrates the cumulative increase in overall dynamic range using 2 dilution steps (1: 2000) at various levels of precision.

For continuous detection over a larger range of concentrations, the dilution factor must be chosen such that the regions of detection from the undiluted and the diluted samples overlap. Also note that if the total number of available partitions is to remain constant (constant available partitioning) between the undiluted and diluted experiment scenarios, making a dilution entails splitting the available number of partitions between the undiluted and diluted samples. Both of these observations demonstrate that the advantage gained (in terms of increased dynamic range) comes at a non-zero cost in terms of the dynamic range of any one concentration and of the lower limit of detection. Fig. 10 shows the necessary overlap of dynamic ranges associated with multiple dilutions as well as the effects that reducing the number of available partitions has on the undiluted sample’s dynamic range and lower limit of detection.

**Fig 11:**
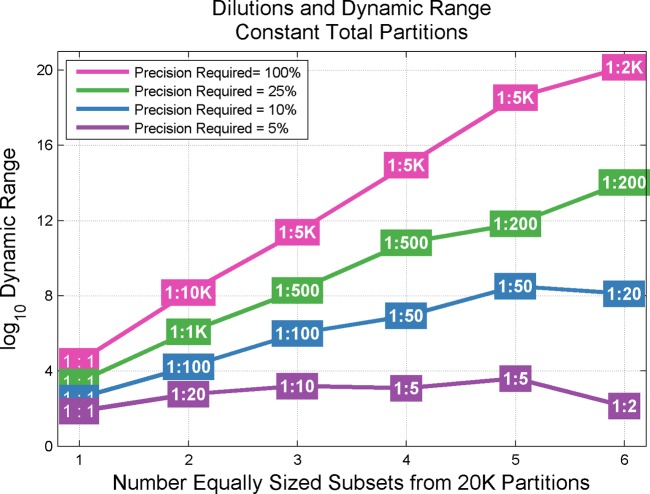
Gains in Dynamic Range via Increasing Number of Dilutions—Constant Available Partitioning. Fig. shows the benefits to dynamic range spanned by increasing the number of dilution steps across available partitioning. X-axis depicts an increasing number of dilution steps spread across a constant (20,160) number of available partitions. Subsets are assumed to be equally sized. As partitions per subset decreases with increasing number of subsets, boxed dilution values represent optimized serial dilutions (# subsets—1 steps) ensuring maximum Dynamic Range gain while maintaining overlapping regions of detection. Benefits to Dynamic Range are slight at high levels of precision (e.g., 5%) and are even seen to occasionally decrease as subsets increase (e.g., 5% precision between 5 and 6 subsets). However, substantial improvement is also seen as precision requirements are relaxed.

**Fig 12:**
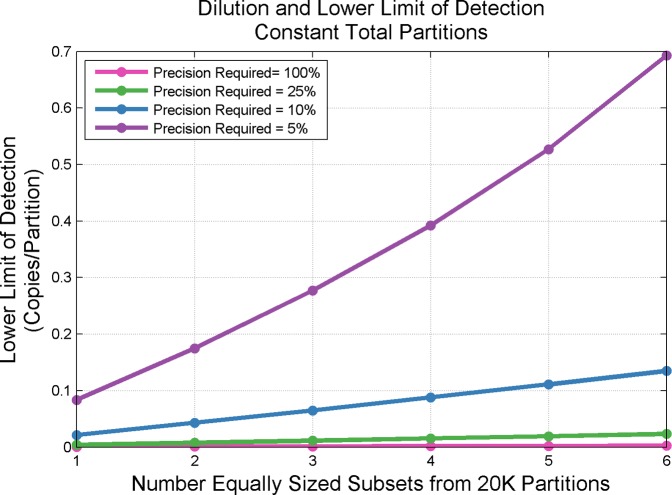
Reduced Sensitivity as the Number of Dilutions Increases—Constant Available Partitioning. Fig. shows the deteriorating sensitivity for the lower limit of detect by increasing the number of dilution steps across available partitioning. Impacts to sensitivity are greatest at high levels of precision (e.g., 5%) but do substantially lessen as precision requirements are relaxed. X-axis depicts an increasing number of dilution steps spread across a constant number (20,160) of available partitions.

Fig. 11 shows the effect of introducing additional dilutions points (again, under constant available partitioning). Additional dilution points beyond a single optimized dilution point do not substantially increase the dynamic range at the 5% precision level. However, as precision requirements are relaxed, substantial gains in dynamic range are seen to occur as the number of dilutions is increased. Such gains in additional dynamic range are conditional upon a willingness to accept elevation of the lower limit of detection (reduced sensitivity) as seen in Fig. 12. Additionally, increasing the number of dilutions may introduce additional sources of variability impacting the effective precision of the system.

**Fig 13:**
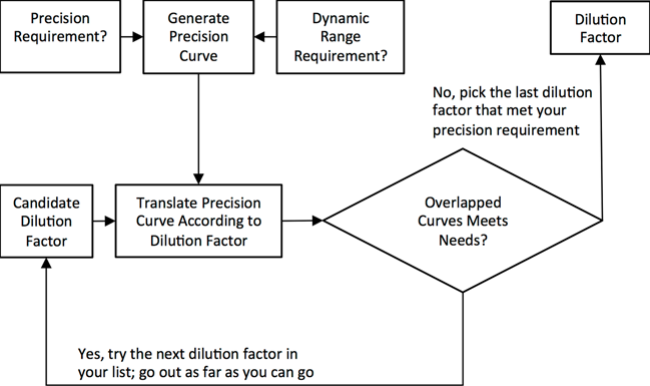
Workflow to Select Dilution Factor For Paired Chip Strategy for Maximizing Dynamic Range. Fig. shows how to select the dilution factor for the second chip in a pairing strategy to maximize the dynamic range.

A paired dilution strategy is devised to demonstrate an effective method for maximizing the available dynamic range. A first chip is run at original concentration. A second chip is run at an appropriate dilution. Fig. 13 shows a workflow that can be used for selection of the dilution factor for the second chip. An automated tool to make these calculations is currently under development.

**Fig 14:**
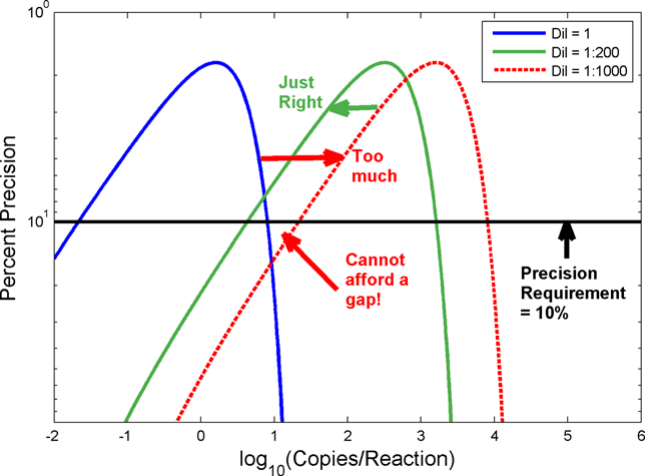
Trials with Two Dilution Factors to Select Optimal One For Use with Paired Dilution Strategy. Fig. shows that the continuous detection criterion fails at the application of the 1:1000 dilution factor. The 1:200 dilution factor meets the continuous detection criterion at a 10% precision requirement.

Fig. 14 demonstrates the translation of the precision curve caused by a candidate dilution factor. It is seen that the continuous detection criterion fails at the application of the 1:1000 dilution factor. The 1:200 dilution factor however meets the continuous detection criterion at a 10% precision requirement.

## Results and Discussion

A digital PCR experiment is conducted highlighting the dilution techniques described. An absolute quantification experiment is designed using a paired dilution strategy to detect reliably across greater than 3 logs of concentration. Digital PCR results are compared against analogous quantitative PCR results running the same original samples

Table 1 shows the sample and assay information used in the experiment.

**Table 1:** Sample, Assay, Reaction Mix and Thermal Protocol Information.

Sample	Human Genomic DNA: Male from Promega (Catalog # G1471): 100 ug (P/N: G147A). This original sample is taken and a 5-point dilution series (1: 6.8) is constructed to yield 5 samples with unknown concentration, labeled as Samples A, B, C, D and E.
Assay used in digital PCR experiment	Taqman Copy Number Assay: Catalog #: 4400291, Assay ID: Hs04107718_cn, Gene Symbol: GLA, RPL36A-HNRNPH2, Assay Location: Chromosome X: 100654057, Scale: S: 360uL; 20X
Assay used in qPCR experiment, results from which are provided for orthogonal validation	Taqman Copy Number Reference Assay RNase P: Catalog #: 4403328. Targets the Ribonuclease P RNA component H1 (H1RNA) gene (RPPH1) on chromosome 14, cytoband 14q11.2. The assay location is chr.14: 20811565 on NCBI build 37. It has an 87 bp amplicon that maps within the single exon RPPH1 gene.
Protocol	The sample was diluted using 1X TE buffer with the following recipe for a 20ul reaction mix: 1ul of sample, 1ul 20X Assay, 10ul 2X Flash Master Mix and 8μl water. The chip was warmed to 30 deg C, and then the entire 20ul reaction was spread across the chip manually, using plastic applicator. Lid was applied and enough degassed Immersion Fluid (included in the kit for preparing the digital PCR chips for QS3D from Thermo Fisher Scientific) was added to fill space between chip and lid. The chips were thermocycled on Dual Flat Block GeneAmp® PCR System 9700 (Thermo Fisher Scientific) at 45deg C, 1min, 98 deg C, 2min 30sec; followed by 40 cycles of 98deg C, 1 min, 56 deg C, 10 sec (30% ramp rate), 60 deg C, 30sec.

Each sample A through E is interrogated on QS3D digital PCR system (Thermo Fisher Scientific) in the following way: Two chips are employed per sample: One at the original concentration, One diluted 1: 50. Three replicates of this two chip setup are run. (Note that running replicates is only to account for pipetting errors and is not necessary for quantification.) The first set of chips per sample has the sample at the original concentration. The second set of chips per sample has the sample diluted 1: 50. The results are analyzed on the QuantStudio 3D AnalysisSuite software (Thermo Fisher Scientific). These data from QuantStudio 3D AnalysisSuite software were exported including detail view of data per chip and is included in S1 data Export and S1 Raw Data Views respectively.

Raw results are then pruned using a set of rules as given in Table 2.

**Table 2:** Rules for Pruning Data in Paired Dilution Experiments for Quantification.

Rule	Effect of applying rule on current dataset
1. Review the NTC (non-template control) chips to ensure that false positive rate for your assay system is acceptable (Typically <50 positives).	Three NTC were run with 4, 13 and 35 positives and thus this criteria was passed.
2. For any chip that is not a green flag, review and manually threshold if needed, rejecting chips if you are not able to see a clear manual threshold.	Manual Thresholds applied (supplemental data provide details for each chip). No chips were rejected.
3. For a given sample, if at least one of the chips is within the preferred concentration range (200–2000 copies/μl), filter out the chips outside of the range.	Rule applied rejecting data points as shown in Fig. 15. The dilute points for samples D and E were not run as we expected results under 200 copies/μl and they would be rejected by Rule 3.
4. If both chips are within the preferred concentration range (200–2000 copies/μl), and are not within 30% of the expected 50X fold dilution discard results.	Rule applied with no chips discarded
5. If all chips are above 4600 copies/ul, use the lowest concentration.	Rule not applied as no paired set of chips were both above 4600 copies/μl
6. If all chips are below 9 copies/μl, use the highest concentration	Rule not applied as no paired set of chips were both under 9 copies/μl

**Fig 15:**
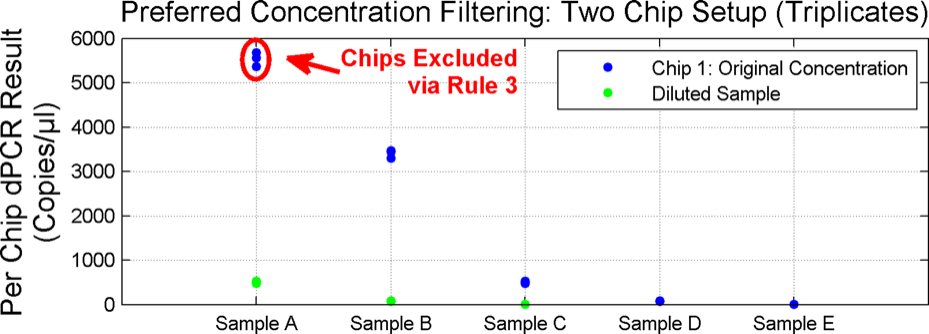
Preferred Concentration Filtering: Two Chip Setup. Fig. shows digital PCR results obtained on the two chip setup and filtered thru Rule 3 for each of 5 Unknown samples (diluted Samples D and E not run). Sample A original concentration chips excluded due to being outside the preferred concentration range (200–2000 copies/μl) while the diluted chips are within the preferred range.

**Fig 16:**
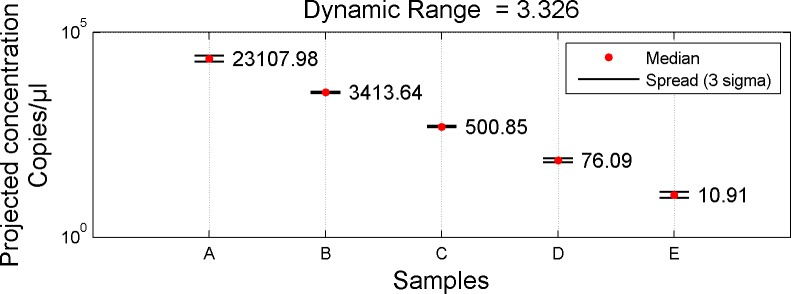
Quantification Results: Two Chip Setup. Fig. shows computed quantification results for the 5 Unknown samples using the two chip strategy. For each Sample, at least one of the chips provided information useful for determining a quantity.

Combining the results across the two dilution points yielded the quantification for each sample as given in Fig. 16.

**Fig 17:**
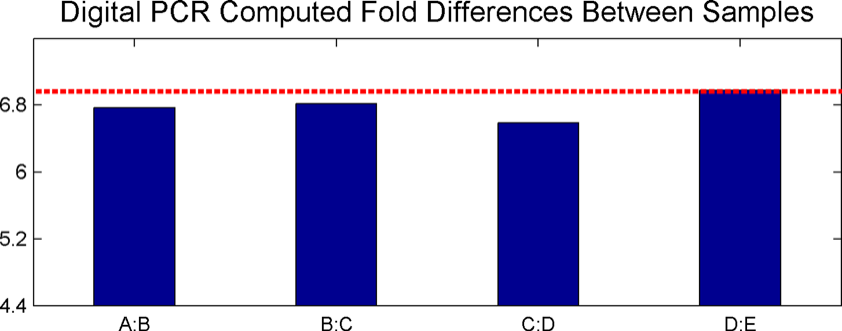
Digital PCR Fold Change between Adjacent Samples. Fig. shows the fold change between digital PCR computed quantities for all adjacent samples (e.g., between Sample A and Sample B). The values are seen to compare very well with the expected 6.8 fold difference between samples.

Computing the fold change between adjacent samples (e.g., between Samples A and B), one finds that the original dilution of 1: 6.8 is well maintained. Fold differences between all adjacent samples are shown in Fig. 17.

**Fig 18:**
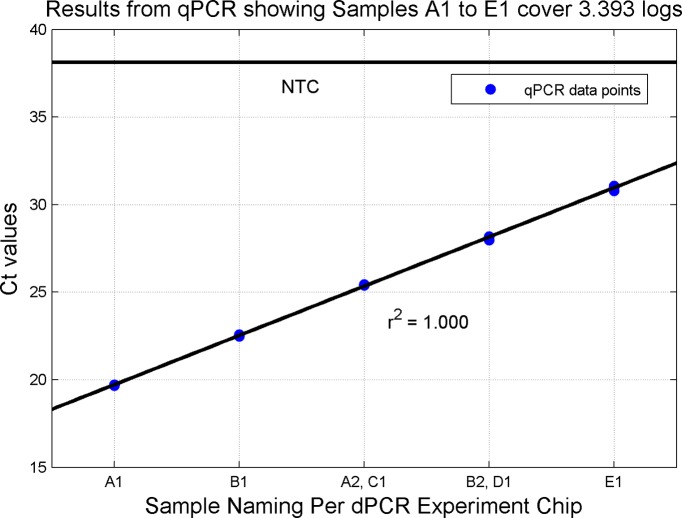
Quantitative PCR C_Q_ Results of Original Dilution Series. Fig. shows computed CQ results for the 5 Unknown samples using the quantitative PCR. The CQ values correspond to a dynamic range of 3.393 logs between Samples A and Sample E and support the digital PCR results.

Quantitative PCR results confirm the fold differences between samples. A separate set of quantitative PCR experiments were run using these same dilution points and the RNase P assay to establish the range of interrogated concentrations using an orthogonal scheme. This result is provided in Fig. 18.

The dynamic range (Sample A to Sample E) demonstrated by the digital PCR experiment computes to approximately 3.326 logs. This compares favorably to the expected range of 3.330 logs based of the original dilution series. The quantitative PCR supports both of these Figs. with a demonstrated range of 3.393 logs.

## Conclusions

### Practical Effects on Precision, Sensitivity, and Dynamic Range

Experimental conditions deviating from theoretical assumptions are seen to have a practical effect on achievable precision and sensitivity for practitioners seeking the benefits offered using digital PCR. Additionally, effective use of dilutions enables an increase in a system’s overall dynamic range as seen in the dilution experiment performed. A summary of the practical effects (and extensibility of dynamic range, via the use of dilutions) on digital PCR is as follows:

- Precision (specifically at higher concentrations) is significantly impacted by volume variation. Peak precision is found to occur at lower concentrations as the amount of volume variation increases. Therefore, in systems with higher volume variability, targeting lower concentrations is preferable.
- Precision is likewise impacted by false reaction call rates. Under non-zero false reaction call rates, it is no longer necessarily desirable to target 20% negatives in order to achieve optimal precision. The direction to target, however, depends on the type of false reaction calls anticipated. As the false positive call rate increases, one would target lower load concentrations. As the false negative call rate increases, one would target higher load concentrations.
- Sensitivity (specifically the lower limit of detection, λ_l_) is significantly impacted by false positive calls. A false positive call rate of 1% is found to raise λ_l_ by an order of magnitude as compared to a 0% false positive call rate.
- Dynamic Range (at a specified precision) can be extended within a set number of partitions via the use of dilutions. Care must be used to identify dilutions optimizing increases in dynamic range while ensuring a continuous ability to detect concentrations throughout the dynamic range (i.e., to prevent gaps in coverage).
- Sensitivity, under a multi dilution scenario with constant partitioning, moderately deteriorates at the lower end of detection but substantially improves at the upper end of detection.
- Dynamic Range, under a multi dilution scenario with constant partitioning, exhibits diminishing returns (particularly at tighter precision requirements) as the number of dilutions increases.

## Supporting Information

S1 data Export Results exported from Thermo Fisher Scientific QuantStudio 3D AnalysisSuite software. (CSV)

S1 Raw Data Views Screen captures from Thermo Fisher Scientific QuantStudio 3D AnalysisSuite software showing details of results from individual digital PCR chips. (DOC)

S1 Appendix Derivation of equations treating dPCR experiments as a binomially distributed random variable. (DOC)
