# Supplementary material for: Digital PCR Modeling for Maximal Sensitivity, Dynamic Range and Measurement Precision
Source: PLoS One. 2015 Mar 25;10(3):e0118833. doi: 10.1371/journal.pone.0118833 (PMC4373789; doi:10.1371/journal.pone.0118833)

This study, created using Thermo Fisher Scientific Quantstudio 3D AnalysisSuite software, represents chips that were run illustrating the dual chip strategy to augment the supported dynamic range of a digital PCR system using paired dilutions. The paper describes samples A - E, run in pairs of original concentration and a dilution point. These are hypothetical samples constructed out of a dilution series experiment, the raw data from which is presented here. Certain chips from this experiment were reused to serve as the concentrated point for one hypothetical sample and the dilution point for another hypothetical sample. The dilution points for samples D and E were not run as they were clearly outside the dynamic range of this system.

**Sample A concentration point**

Sample A1_conc Sample A2_conc


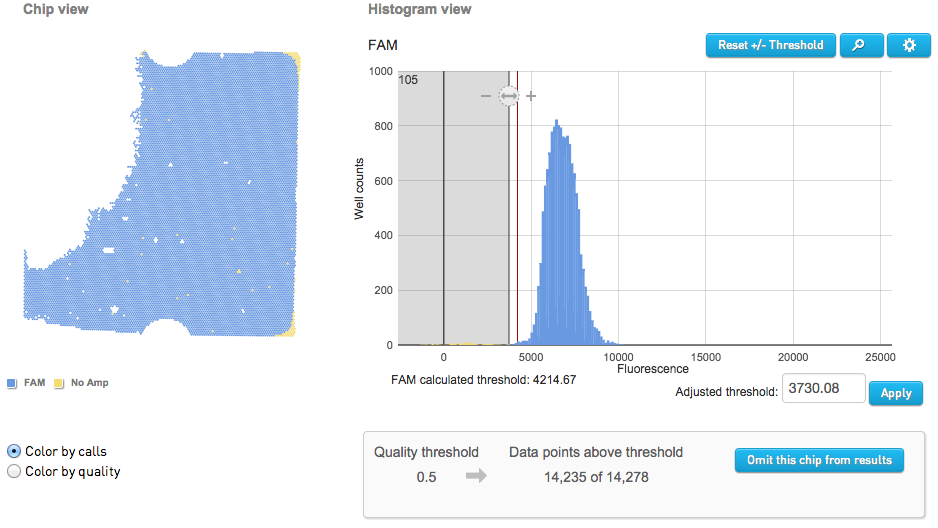

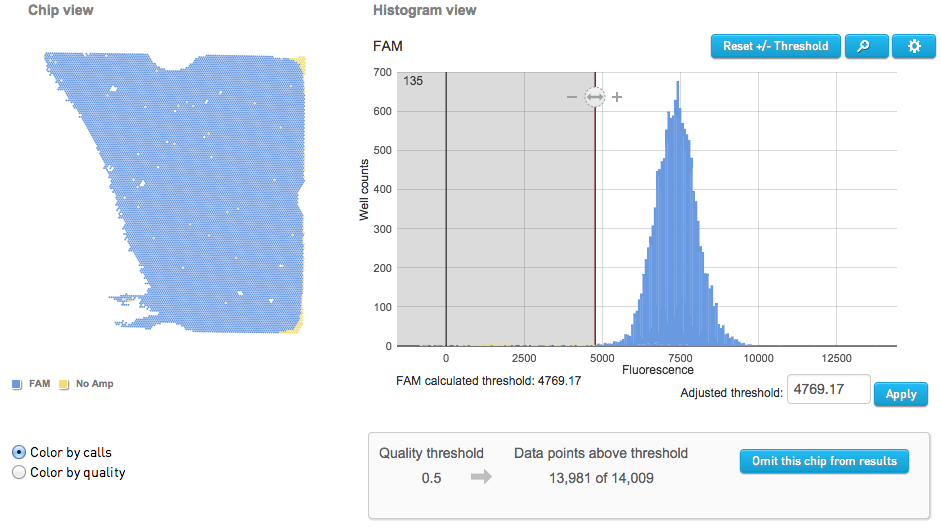


Sample A3_conc


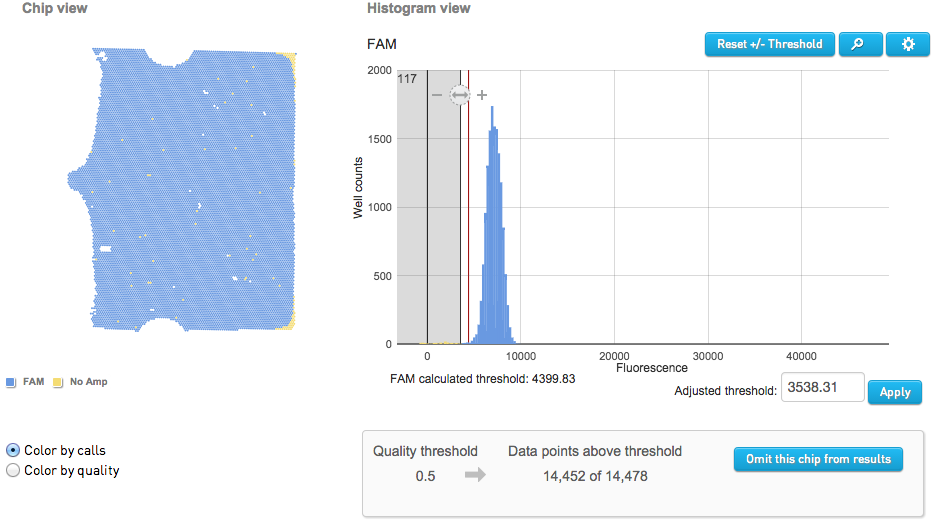


Sample A concentration points were all filtered out by applying rule 3 as explained in the paper.

**Sample A dilution point**

Sample C1_conc, A1_dil Sample C2_conc, A2_dil


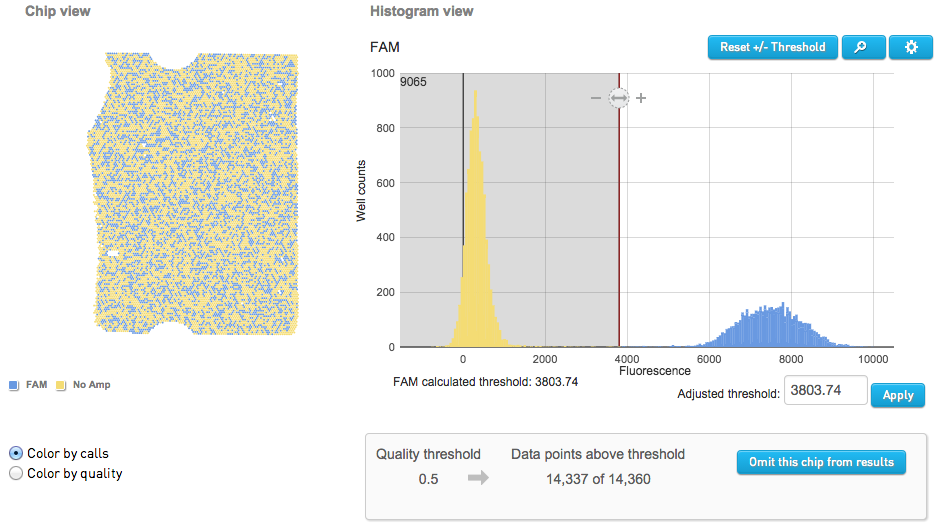

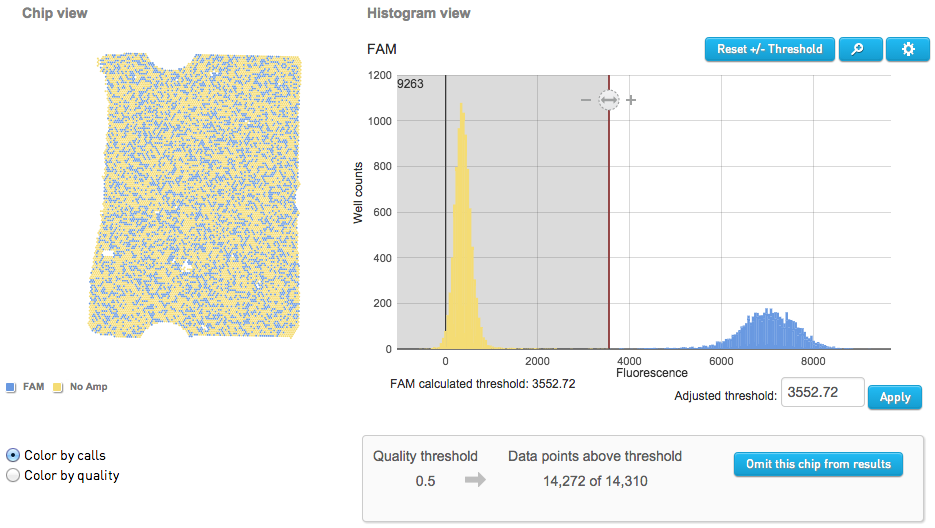


Sample C3_conc, A3_dil


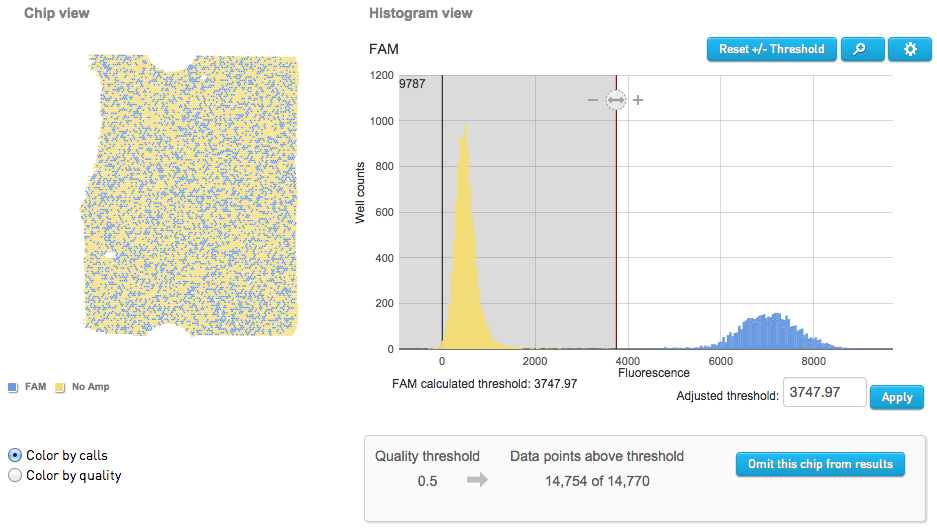


**Sample B concentration point**

Sample B1_conc Sample B2_conc


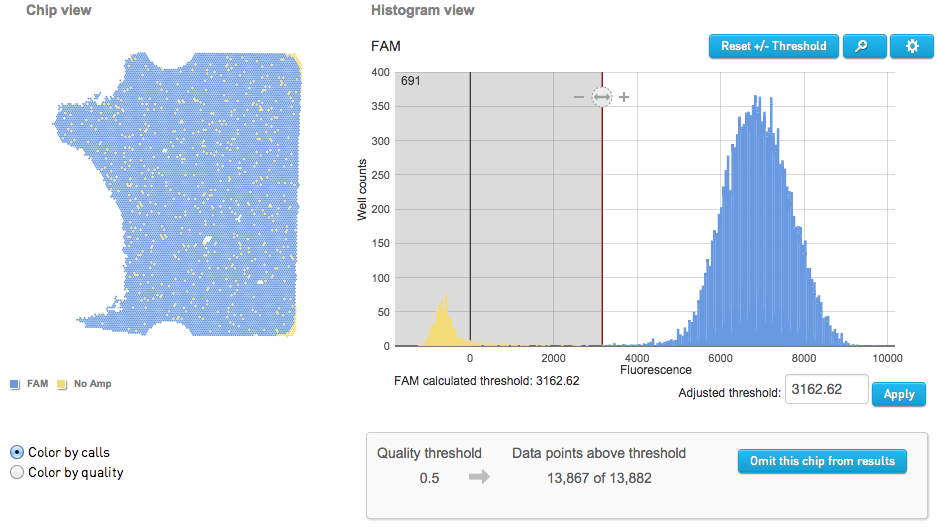

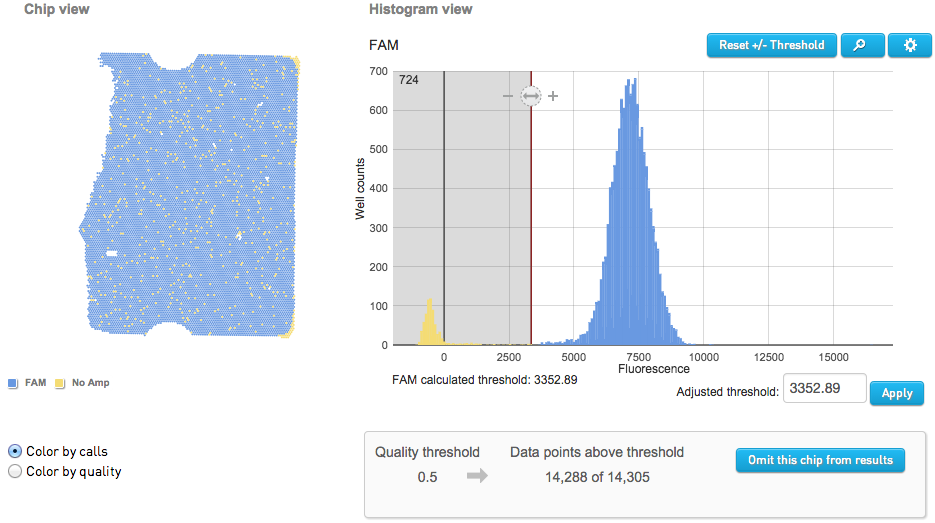


Sample B3_conc


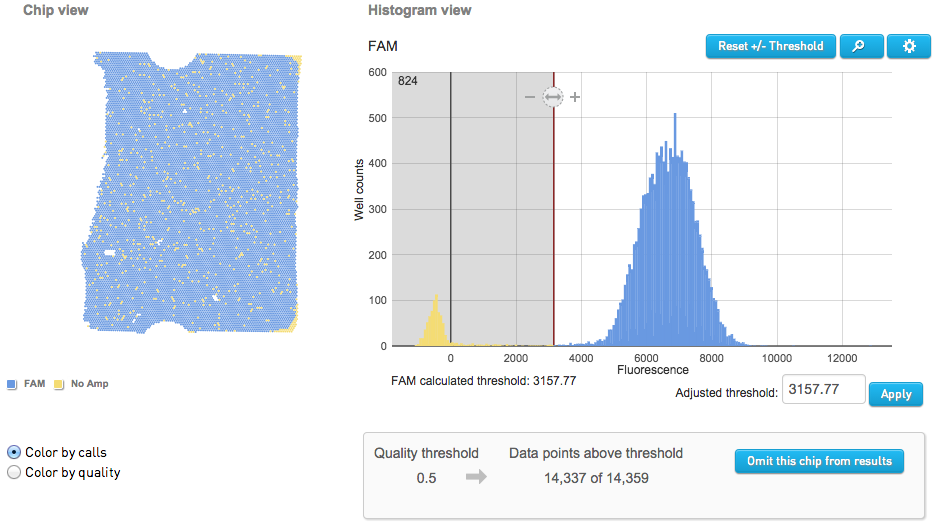


**Sample B dilution point**

Sample D1_conc, B1_dil Sample D2_conc, B2_dil


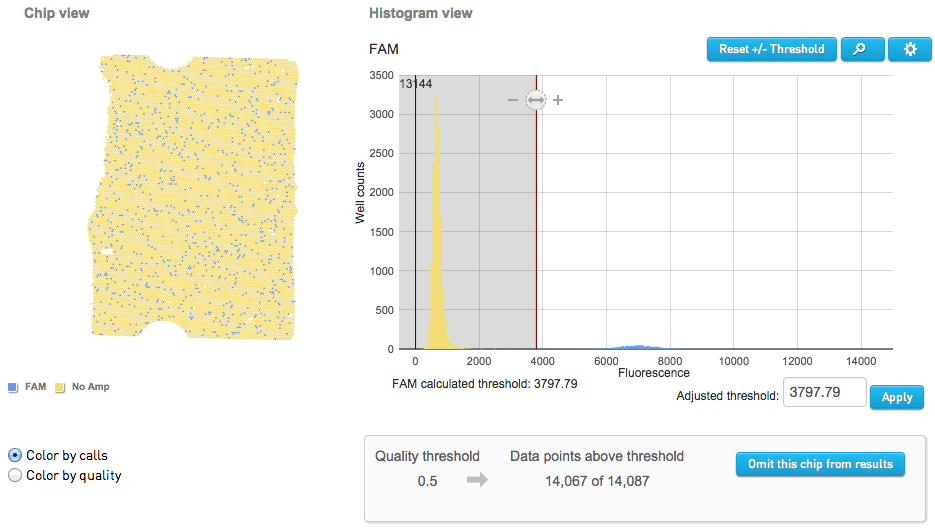

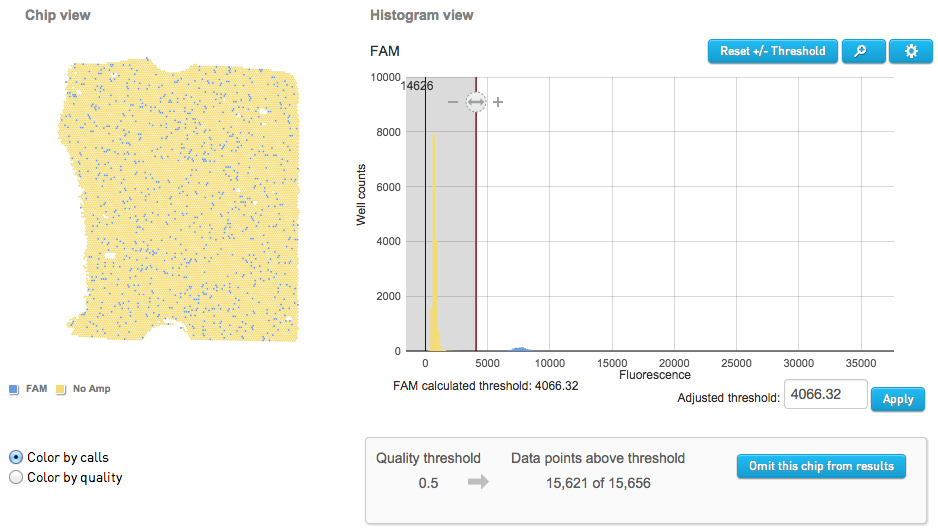


Sample D3_conc, B3_dil


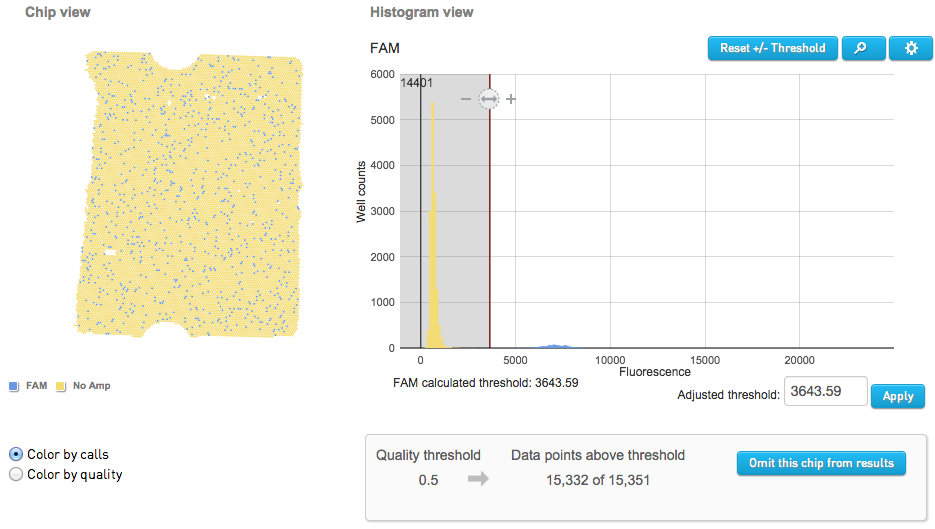


**Sample C concentration point**

Sample C1_conc, A1_dil Sample C2_conc, A2_dil


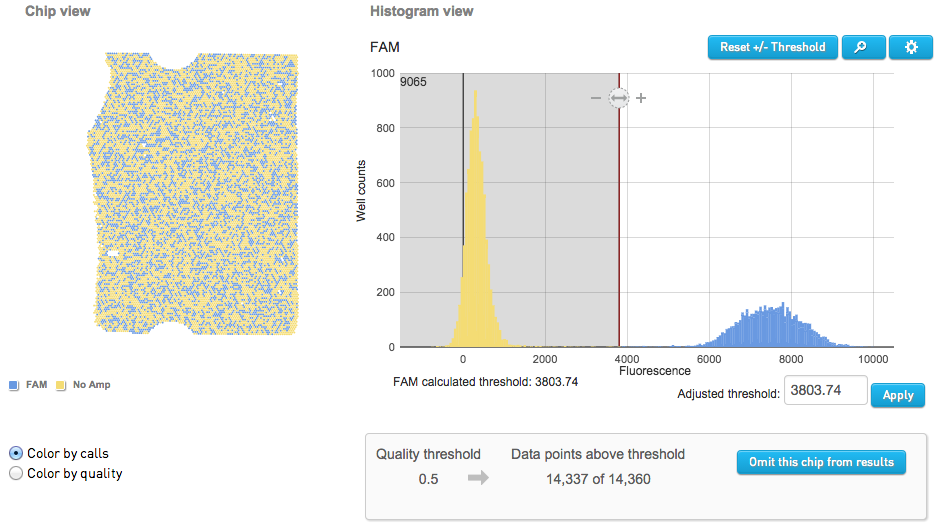

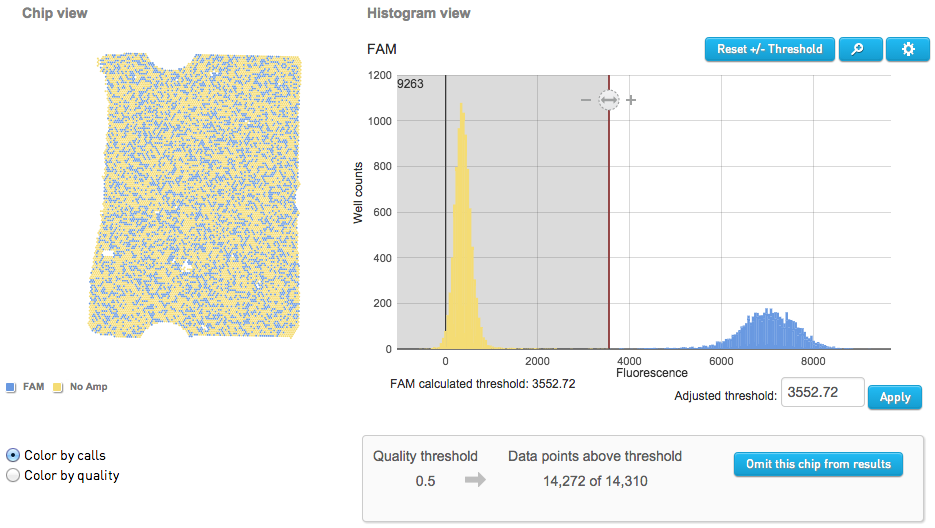


Sample C3_conc, A3_dil


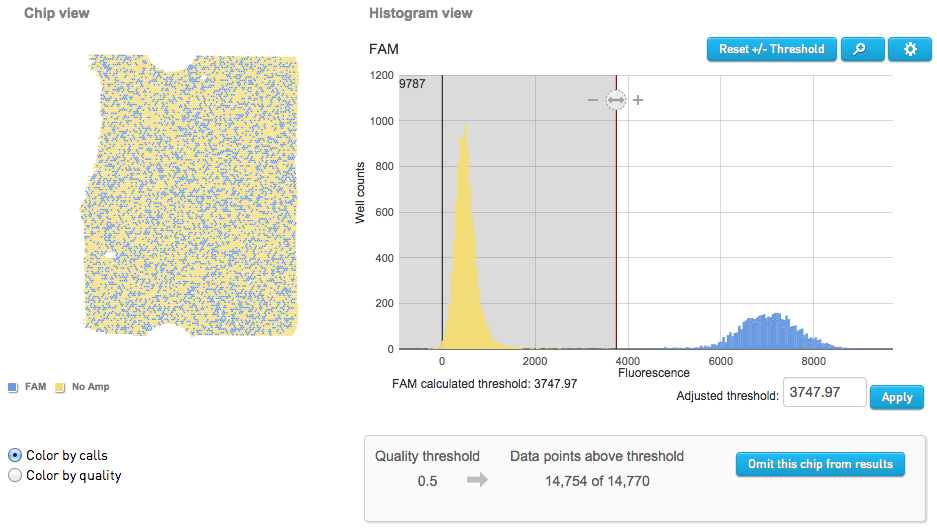


**Sample C dilution point**

Sample E1_conc, C1_dil Sample E2_conc, C2_dil


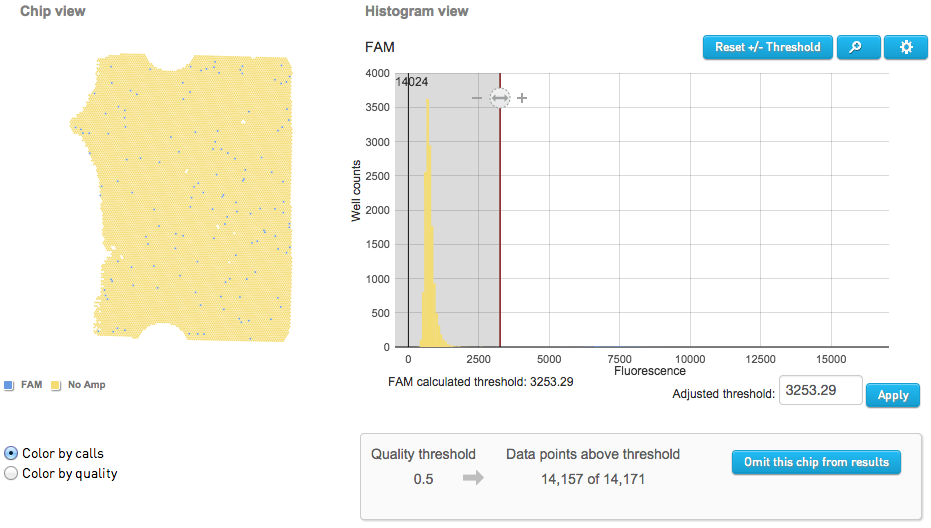

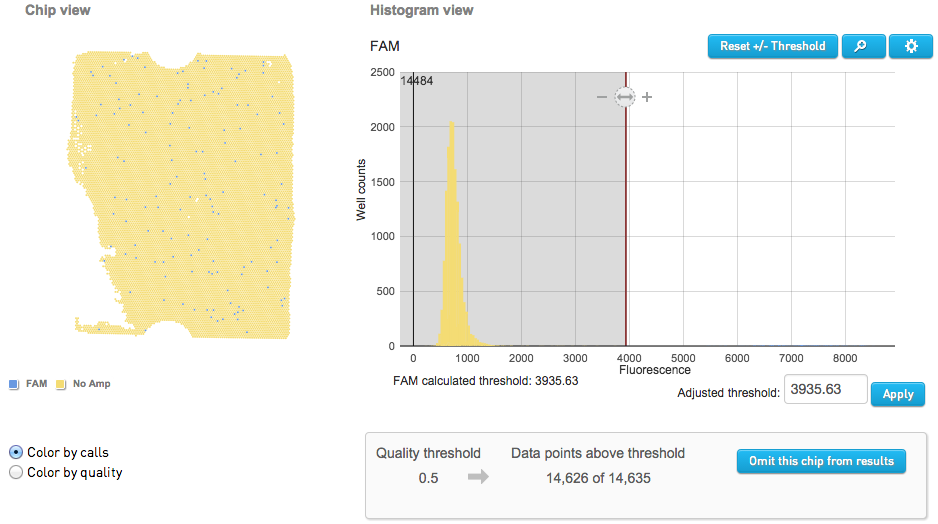


Sample E3_conc, C3_dil


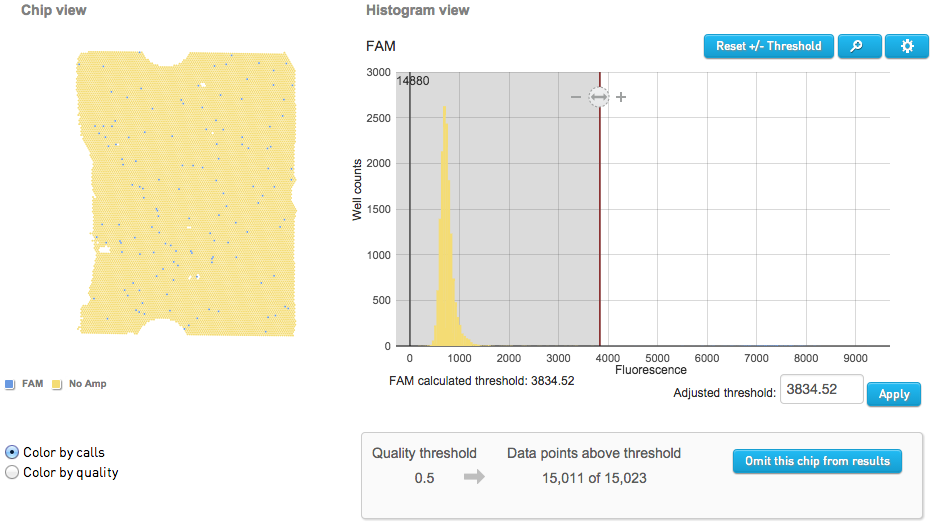


**Sample D concentration point**

Sample D1_conc, B1_dil Sample D2_conc, B2_dil


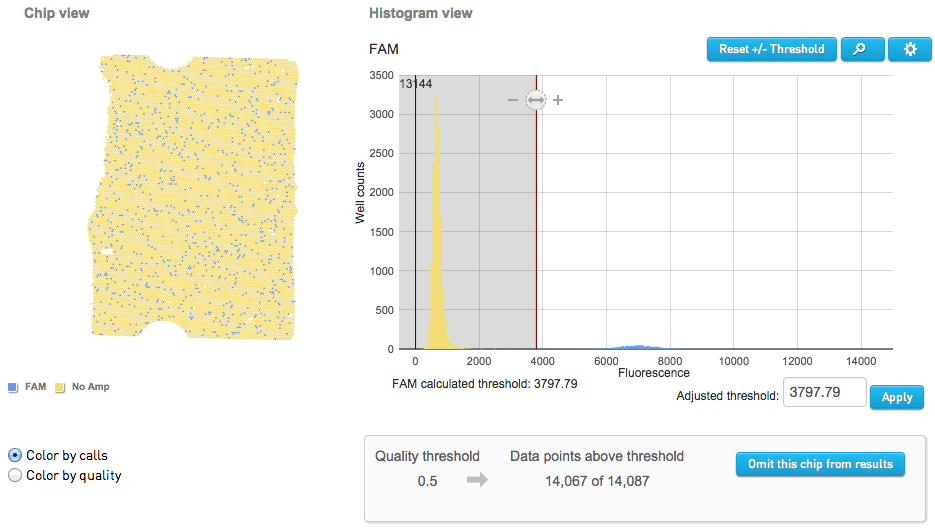

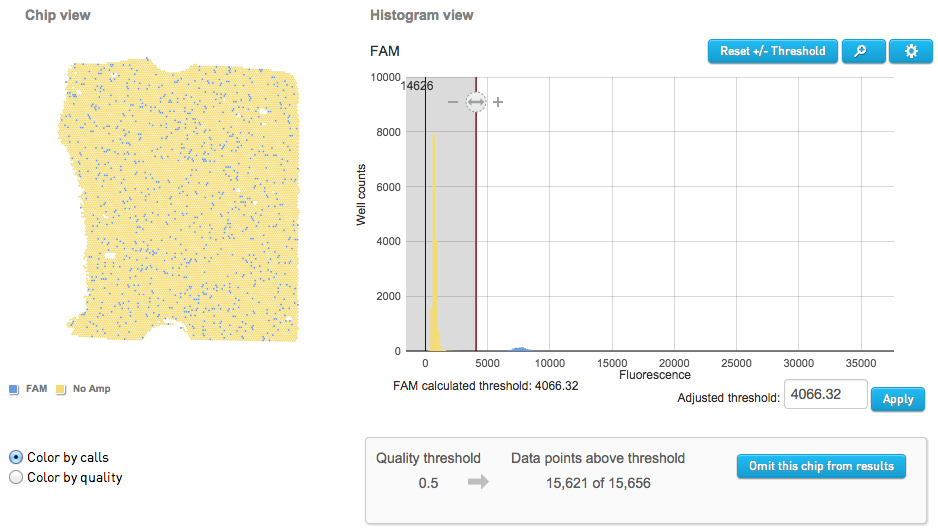


Sample D3_conc, B3_dil


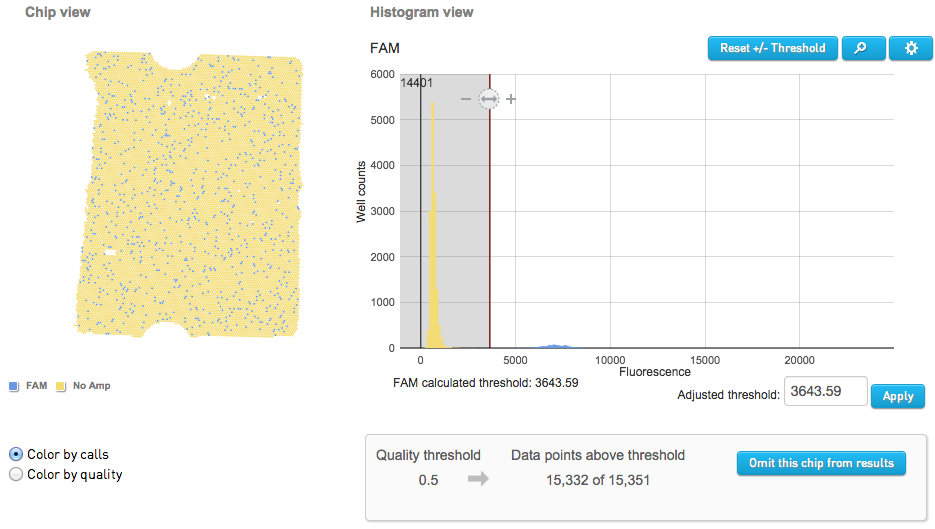


Dilution points for Sample D not run as they were clearly outside dynamic range.

**Sample E concentration point**

Sample E1_conc, C1_dil Sample E2_conc, C2_dil


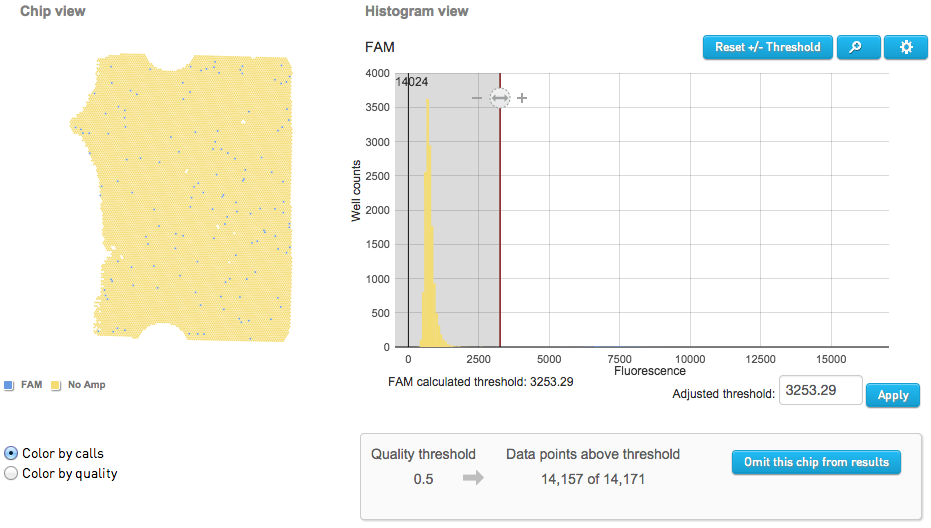

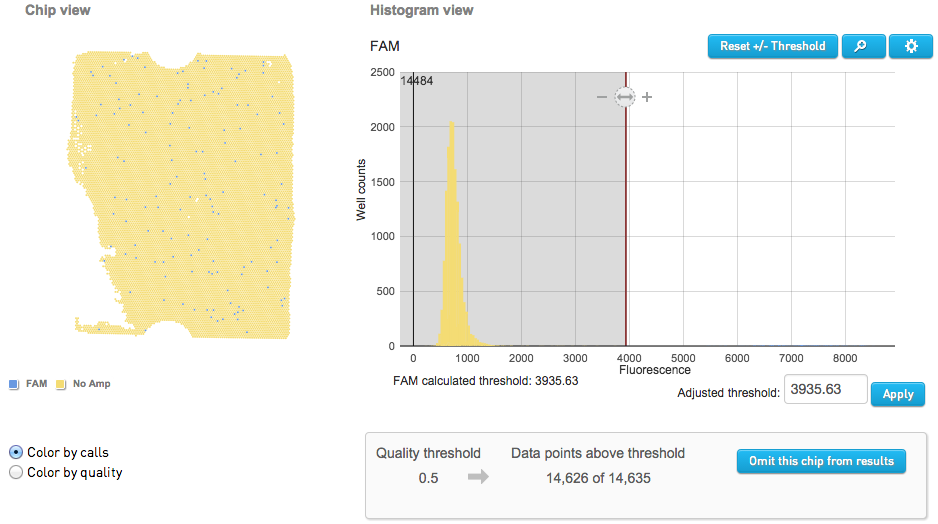


Sample E3_conc, C3_dil


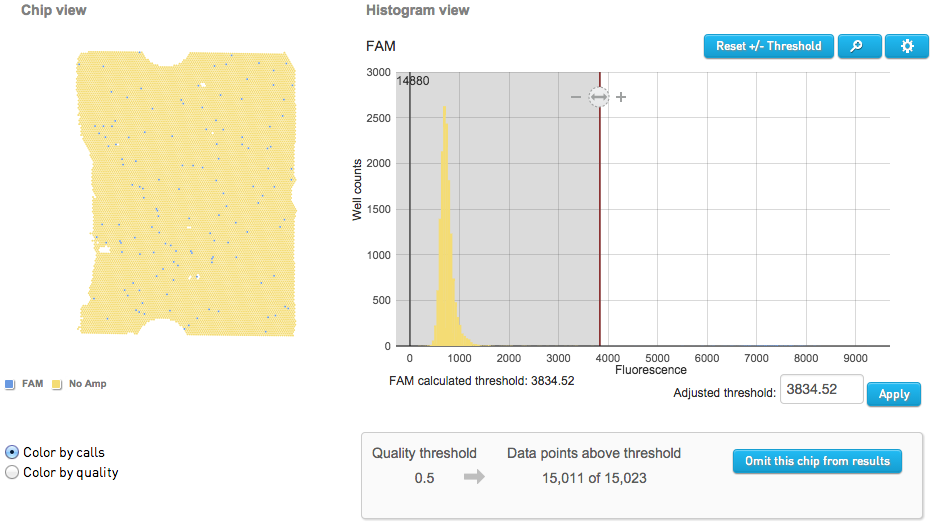


Dilution points for Sample E not run as they were clearly outside dynamic range.

**NTC**

NTC 1 NTC2


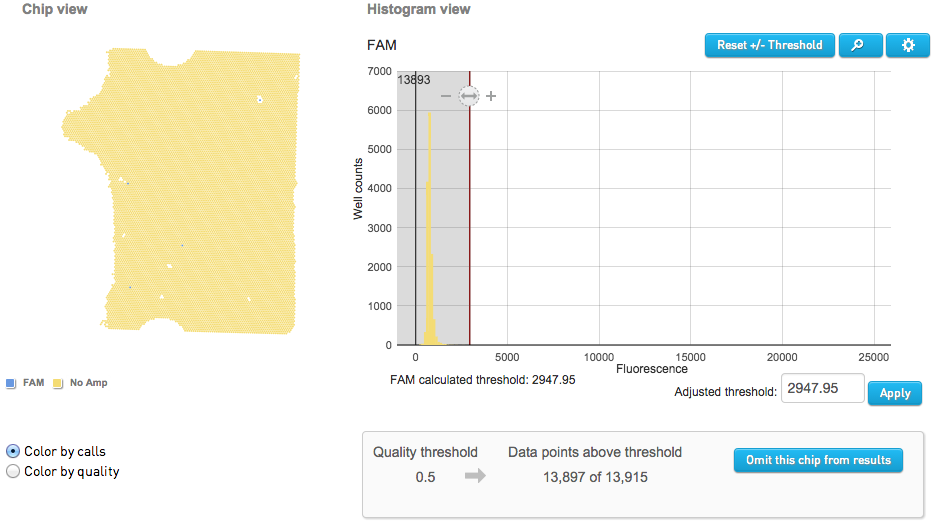

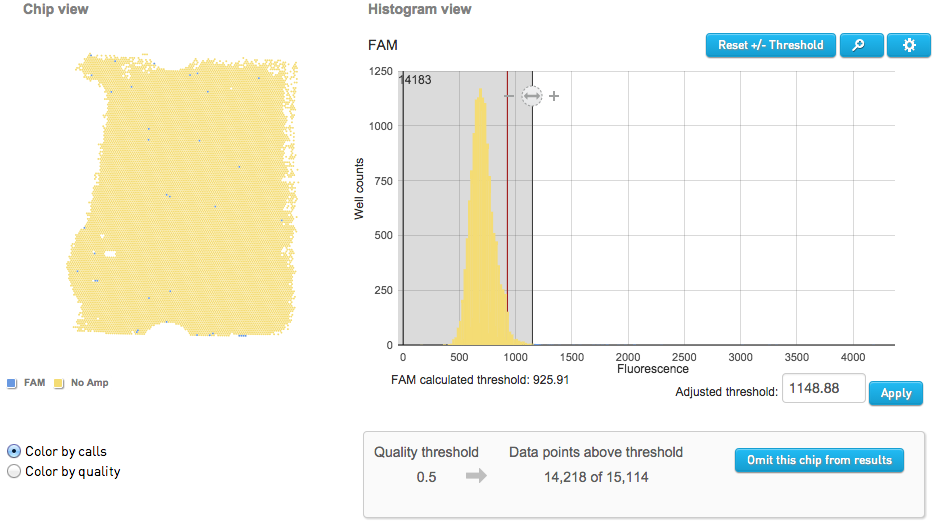


NTC3


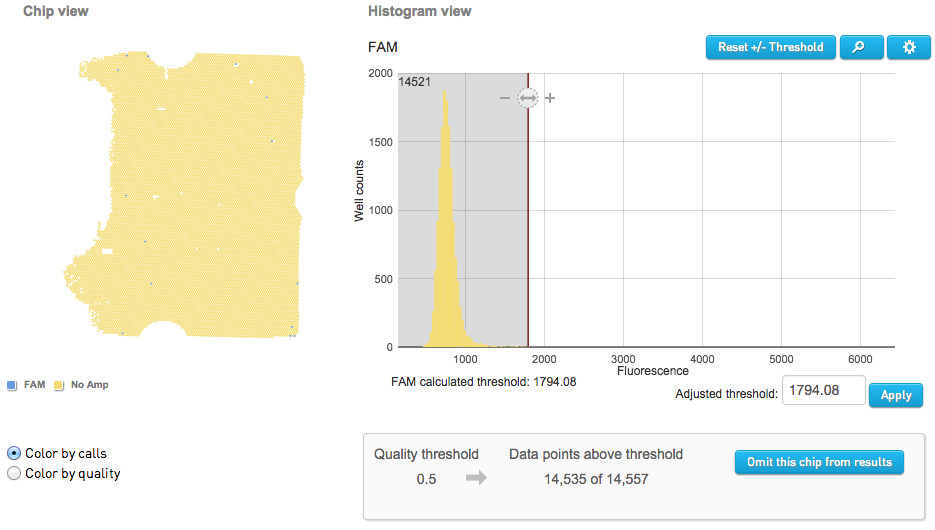

Supplement: S1 Raw Data Views — (DOC) [file pone.0118833.s002.doc]
