# Supplementary material for: Digital PCR Modeling for Maximal Sensitivity, Dynamic Range and Measurement Precision
Source: PLoS One. 2015 Mar 25;10(3):e0118833. doi: 10.1371/journal.pone.0118833 (PMC4373789; doi:10.1371/journal.pone.0118833)
Supplement: S1 Appendix — (DOC) [file pone.0118833.s003.doc]

## Appendix A: Derivation of Lower and Upper Limits of Detection Treating dPCR Experiments as Binomial Distributed Random Variables

Consider treating each of the partitions as an independent Bernoulli random variable. We define success (p) somewhat non-intuitively as the probability of the partition containing zero target molecules, and we define failure (q) as the probability of the partition containing one or more target molecules. These probabilities are represented, via Poisson probabilities, as

$$\begin{matrix} p=e^{-\lambda} \\ q=\left(1-e^{-\lambda}\right) \end{matrix}$$

Since the partitions are independent, their sum (or, alternatively, the experiment itself) defines a Binomial distributed random variable X. The probability mass function of a Binomial distribution is given as follows:

$$P\left\{X=x|n,p\right\}=\left(\begin{matrix} n \\ x \end{matrix}\right)p^{x}q^{n-x}$$

Conceptually, these limits are the concentrations that achieve zero and n positives partitions respectively at a given a desired confidence level of detection ($1-\alpha$). This can be expressed via the following expressions:

$$P\left\{X=n|n,p\right\}=\left(\begin{matrix} n \\ n \end{matrix}\right)p^{n}q^{n-n}=p^{n}=e^{-\lambda_{l}n}$$

$$P\left\{X=0|n,p\right\}=\left(\begin{matrix} n \\ 0 \end{matrix}\right)p^{0}q^{n-0}=q^{n}=\left(1-e^{-\lambda_{u}}\right)^{n}$$

Setting these expressions equal to the desired level of confidence we have:

$$e^{-\lambda_{l}n}=\left(1-\alpha\right)$$

$$\left(1-e^{-\lambda_{u}}\right)^{n}=\left(1-\alpha\right)$$

Solving for the limits produces the expressions desired:

$$\lambda_{l}=-\ln\left(1-\alpha\right)^{\frac{1}{n}}$$

$$\lambda_{u}=-\ln\left(1-\left(1-\alpha\right)^{\frac{1}{n}}\right)$$
